# Effectiveness and Safety of DOACs vs. VKAs in AF Patients With Cancer: Evidence From Randomized Clinical Trials and Observational Studies

**DOI:** 10.3389/fcvm.2021.766377

**Published:** 2021-11-05

**Authors:** Fuwei Liu, Zixuan Xu, Jun Luo, Peng Yu, Jianyong Ma, Ping Yuan, Wengen Zhu

**Affiliations:** ^1^Department of Cardiology, The Affiliated Ganzhou Hospital of Nanchang University, Ganzhou, China; ^2^Department of Emergency, The Third Affiliated Hospital of Sun Yat-sen University, Guangzhou, China; ^3^Department of Endocrinology and Metabolism, The Second Affiliated Hospital of Nanchang University, Nanchang, China; ^4^Department of Pharmacology and Systems Physiology, University of Cincinnati College of Medicine, Cincinnati, OH, United States; ^5^Department of Cardiology, The Second Affiliated Hospital of Nanchang University, Nanchang, China; ^6^Department of Cardiology, The First Affiliated Hospital of Sun Yat-sen University, Guangzhou, China

**Keywords:** atrial fibrillation, cancer, direct oral anticoagulants, vitamin K antagonists, meta-analysis

## Abstract

**Background:** The use of direct oral anticoagulants (DOACs) is recommended as the preferred treatment drug in patients with nonvalvular atrial fibrillation (AF). However, the effectiveness and safety of DOACs compared with vitamin K antagonists (VKAs) in patients with cancer and AF are still controversial. Therefore, we performed a meta-analysis regarding the effectiveness and safety of DOACs vs. VKAs in AF patients with cancer.

**Methods:** A search of the Pubmed and EMBASE databases until August 2021 was performed. Adjusted risk ratios (RRs) and 95% confidence intervals (CIs) were pooled using a random-effects model with an inverse variance method.

**Results:** Thirteen studies were deemed to meet the criteria. For the effectiveness outcomes, the use of DOACs compared with VKAs use was significantly associated with decreased risks of stroke or systemic embolism (RR = 0.66, 95% CI: 0.54–0.80) and venous thromboembolism (RR = 0.40, 95% CI: 0.26–0.61), but not ischemic stroke (RR = 0.79, 95% CI: 0.56–1.11), myocardial infarction (RR = 0.78, 95% CI: 0.56–1.11), cardiovascular death (RR = 0.76, 95% CI: 0.53–1.09), and all-cause death (RR = 0.82, 95% CI: 0.43–1.56). For the safety outcomes, compared with VKAs use, the use of DOACs was associated with reduced risks of intracranial bleeding (RR = 0.60, 95% CI: 0.50–0.71) and gastrointestinal bleeding (RR = 0.87, 95% CI: 0.80–0.95). There were no significant differences in major bleeding (RR = 0.87, 95% CI: 0.74–1.04), major or nonmajor clinically relevant bleeding (RR = 0.87, 95% CI: 0.74–1.01), and any bleeding (RR = 0.88, 95% CI: 0.76–1.03).

**Conclusion:** Compared with VKAs, DOACs appeared to have significant reductions in stroke or systemic embolism, venous thromboembolism, intracranial bleeding, and gastrointestinal bleeding, but comparable risks of ischemic stroke, myocardial infarction, cardiovascular death, all-cause death, major bleeding, major or nonmajor clinically relevant bleeding, and any bleeding in patients with AF and cancer.

## Introduction

Atrial fibrillation (AF) is the most common arrhythmia in adults. The currently estimated prevalence of AF in adults is between 2 and 4%, and a 2.3-fold rise is expected, due to the longevity in the general population and the increased screenings of patients with undiagnosed AF (1). Increasing age is a foremost risk factor, but the increasing burdens of other comorbidities (e.g., hypertension, diabetes mellitus, heart failure, coronary artery disease, chronic kidney disease) are also important. Several other modifiable risk factors are potential contributors to AF development and progression (1). AF increases the risks of cardiovascular and cerebrovascular complications including a 5-fold risk of stroke (2). AF-related thromboembolic events are the main reasons for the increased rates of morbidity and mortality (3, 4).

A published research report involving more than 24,000 patients diagnosed with cancer showed that the prevalence of AF combined at the time of cancer diagnosis was about 2.4%, and the incidence of AF after cancer diagnosis was 1.8% (5). AF and cancer may interact with each other on pathophysiological grounds. AF in cancer patients may be caused by inflammation, age, comorbidities, surgery or medical cancer treatment, or direct tumor effects. However, cancer patients are at higher risks of thromboembolism and bleeding complications, because cancer interacts with the coagulation system, which is related to a hypercoagulable state (2). AF and cancer have independently increased risks of arterial and venous thrombosis compared with a single disease. Anticoagulation therapy for patients with AF and cancer is challenging because of the increased risk of thromboembolism and bleeding in this special population.

The current international guidelines recommend the use of direct oral anticoagulants (DOACs) as replacement therapy for vitamin K antagonists (VKAs) in patients with nonvalvular AF. DOACs also have advantages in the elderly, or AF patients with specific diseases such as acute coronary syndrome and chronic kidney disease (6). However, whether these recommendations apply to patients with cancer and AF needs further evidence. So far, most of the data on anticoagulant therapy for cancer patients is mainly for the treatment and prevention of venous thromboembolism (VTE). International guidelines recommend low molecular weight heparin (LMWH) (rather than VKAs or DOACs) for the prevention and treatment of VTE in cancer patients (7). Although DOACs have non-inferiority compared with VKAs in patients with AF, these drugs are not recommended in the guidelines for cancer patients. The effectiveness and safety of anticoagulation therapy in patients with AF and cancer are unclear.

Previous DOAC-related randomized controlled trials (RCTs) in the AF population only include a small number of cancer patients or even exclude some cancer patients (8–11). Current data of *post-hoc* analyses of RCTs (12–15) and observational cohort studies (3, 16–19) regarding the effectiveness and safety of DOACs compared with VKAs in patients with AF and cancer have been published. Therefore, this meta-analysis aimed to evaluate the effect of DOACs vs. VKAs in AF and cancer patients.

## Methods

### Literature Retrieval

The two common databases of PubMed and Embase were systematically searched until August 2021 for available studies using the following search terms: (1) atrial fibrillation, (2) cancer OR tumor OR malignancy, (3) non-vitamin K antagonist oral anticoagulants OR direct oral anticoagulants OR dabigatran OR rivaroxaban OR apixaban OR edoxaban, and (4) vitamin K antagonists OR warfarin. The detailed searching strategies are shown in Supplementary Table 1. In this meta-analysis, we included publications in English.

### Inclusion and Exclusion Criteria

We included the *post-hoc* analyses of RCTs or observational cohort studies focusing on the effectiveness and/or safety of DOACs (dabigatran, rivaroxaban, apixaban, or edoxaban) compared with VKAs in AF patients with cancer. The effectiveness outcomes included stroke or systemic embolism (SSE), ischemic stroke, myocardial infarction (MI), VTE, all-cause death, cardiovascular death; whereas the safety outcomes included major bleeding, major or nonmajor clinically relevant (NMCR) bleeding, intracranial bleeding, gastrointestinal bleeding, and any bleeding. The follow-up time was not restricted. We excluded certain publication types such as reviews, case reports, case series, editorials, and meeting abstracts because they had no sufficient data. Studies with overlapping data were also excluded.

### Study Screenings and Data Extraction

Two authors (FW-L and ZX-X) independently did the process of data extraction. We first screened the titles and abstracts of the searched records to select potential studies, and the full text of which was screened in the subsequent phase. Disagreements were resolved through discussion, or consultation with the third researcher (WG-Z). If two or more studies were from the same data source, the study that was more designed to meet the predefined criteria was included. If two studies met the inclusion criteria, we would include the newly published study, or the study with the longest follow-up or highest sample size.

Two authors independently collected the following characteristics from each included study, mainly included the first author and publication year, location, data source, study design, inclusion period, patient age and sex, types of DOACs, follow-up time, effectiveness and safety outcomes, type of cancers, the sample size and number of events in the VKA- or DOAC- groups, and adjusted risk ratios (RRs) and 95% confidence intervals (CIs).

### Study Quality Assessment

Two authors (FW-L and ZX-X) used the Newcastle-Ottawa Scale (NOS) to perform the quality assessment for the included studies independently. The NOS tool had three domains with a total of nine points including the selection of cohorts (0–4 points), the comparability of cohorts (0–2 points), and the assessment of the outcomes (0–3 points). In this study, we defined studies with the NOS of <6 points as low quality (20).

### Statistical Analysis

We assessed the consistency across the included studies using the Cochrane *Q*-test and *I*^2^ statistic. A *P* < 0.1 for the Q statistic, or *I*^2^ ≥ 50% indicated substantial heterogeneity. We first collected the sample size and number of events in the VKA- or DOAC-groups and calculated their corresponding crude rates of effectiveness and safety outcomes. The comparison results between the VKA- or DOAC-groups were expressed as odds ratios (ORs) and 95% CIs. Second, we assessed the effectiveness and safety of DOACs vs. VKAs in AF patients with cancer using the adjusted RRs. The adjusted RRs and 95% CIs were converted to the natural logarithms and standard errors, which were pooled by a random-effects model using an inverse variance method. The publication bias for the reported effect estimates was assessed using the funnel plots.

All the statistical analyses were conducted using the Review Manager Version 5.3 (The Nordic Cochrane Center, The Cochrane Collaboration, 2014, Copenhagen, Denmark; https://community.cochrane.org/). The statistical significance threshold was set at a *P* < 0.05.

## Results

The process of the literature retrieval is presented in Supplementary Figure 1. A total of 1,116 studies were identified through the electronic searches in the PubMed and Embase databases. According to the predefined criteria, we finally included 13 studies (four *post-hoc* analyses of RCTs and nine observational cohorts) in this meta-analysis (3, 4, 12–17, 19, 21–24). Table 1 shows the baseline patient characteristics of the included studies. All of these included studies had a moderate-to-high quality with the NOS score of ≥6 points.

**Table 1:** Baseline characteristics of the included studies.

**Included studies**	**Study design**	**Data source**	**Sample size**	**Age (mean, y)/Sex**	**DOACs**	**VKAs**	**Efficacy outcomes**	**Safety outcomes**	**Follow-up (years)**	**Types of cancers**
Chen et al. (12)	*Post-hoc* analysis of RCT	ROCKET AF; multicenter	640	77/both	Rivaroxaban	Warfarin	SSE, ischemic stroke, VTE, MI, cardiovascular death, and all-cause death	Major bleeding, major or NMCR bleeding, intracranial bleeding, and any bleeding	1.9	Prostate (28.6%), breast (14.7%), gastrointestinal (3.0%), lung (3.1%), head and neck (3.9%), colorectal (16.1%), melanoma (5.9%), leukemia or lymphoma (5.2%), gynecologic (6.6%), genitourinary (12.2%), thyroid (2.5%), brain (0.3%), unspecified (3.9%), and others (3.0%)
Fanola et al. (13)	*Post-hoc* analysis of RCT	ENGAGE AF-TIMI 48; multicenter	1,153	75/both	Edoxaban	Warfarin	SSE, ischemic stroke, MI, cardiovascular death, and all-cause death	Major bleeding, major or NMCR bleeding, and any bleeding	2.8	Prostate (13.7%), breast (6.5%), bladder (7.5%), gastrointestinal (20.5%), lung or pleura (11.0%), skin (5.9%), liver, gallbladder, or bile ducts (3.8%), pancreatic (3.8%), esophageal (2.5%), renal (2.5%), uterine (2.1%), oropharyngeal (2.6%), brain (2.1%), genital (1.3%), thyroid (1.1%), leukemia (2.8%), lymphoma (2.2%), others (1.3%), and unspecified (1.5%)
Melloni et al. (14)	*Post-hoc* analysis of RCT	ARISTOTLE; multicenter	1,236	–/both	Apixaban	Warfarin	SSE, ischemic stroke, VTE, MI, and all-cause death	Major bleeding, major or NMCR bleeding, intracranial bleeding, and any bleeding	1.8	Prostate (29%), breast (16%), bladder (7%), colon (11%), gastric (2%), ovarian/uterus (6%), lung (3%), melanoma (6%), rectal (3%), renal cell carcinoma (4%), Hodgkin's lymphoma (1%), non-Hodgkin's lymphoma (1%), leukemia (<1%), lymphoma (1%), and others (10%)
Flack et al. (15)	Observational cohort	RE-LY; multicenter	546	–/both	dabigatran	Warfarin	–	Gastrointestinal bleeding	2.2	Gastrointestinal
Ording et al. (16)	Observational cohort	Danish population-based medical databases	11,855	77/both	Not available	Unspecified	Ischemic stroke, VTE, and MI	Gastrointestinal bleeding	1.0	Urological (15%), breast cancer (12%), gastrointestinal (12%), lung (4%), hematological (3%), intracranial (0.1%), and others (54%)
Ording et al. (22)	Observational cohort	Danish nationwide cohort study	1,476	78/both	Dabigatran, rivaroxaban, apixaban, and edoxaban	Unspecified	–	Intracranial bleeding, gastrointestinal bleeding, and any bleeding	1.0	Gastrointestinal
Shah et al. (3)	Observational cohort	Market Scan databases, the United States	16,096	74/both	Dabigatran, rivaroxaban, and apixaban	Warfarin	Ischemic stroke, VTE	Any bleeding	1.0	Breast (19.2%), gastrointestinal (12.7%), lung (12.3%), Genitourinary (29.2%), gyneco-oncological (2.4%), hematological (9.8%), and others (14.4%)
Kim et al. (4)	Observational cohort	Severance Cardiovascular Hospital, Seoul, Korea	1,651	70/both	Dabigatran, rivaroxaban, and apixaban	Warfarin	SSE, all-cause death	Major bleeding, intracranial bleeding, and gastrointestinal bleeding	1.8	Prostate (9.3%), gastrointestinal (20.6%), breast (2.4%), colorectal (14.9%), thyroid (10.8%), lung (12.2%), melanoma (5.9%), biliary tract (5.4%), urinary tract (6.1%), genitourinary (12.2%), head and neck (4.1%), hepatocellular carcinoma (3.0%), ovary and endometrial (2.6%), renal cell carcinoma (3.1%), hematologic malignancy (2.2%), and others (3.2%)
Pardo Sanz et al. (23)	Observational cohort	AMBER-AF registry, Oncology and Cardiology Departments, Spain	637	75.4/Female	Not available	Unspecified	SSE	Major bleeding	2.8	Breast
Sawant et al. (17)	Observational cohort	The national VA Healthcare data	196,521	76/both	dabigatran, rivaroxaban, apixaban	Warfarin	Ischemic stroke, all-cause death	NA	1.0	Not available
Yasui et al. (19)	Observational cohort	Osaka International Cancer Institute, Japan	224	72.7/both	Dabigatran, rivaroxaban, apixaban, and edoxaban	Warfarin	SSE, ischemic stroke	Major bleeding, intracranial bleeding, gastrointestinal bleeding	1.0	Gastrointestinal (44.2%), Lung (24.1%), genitourinary (11.2%), head and neck (9.8%), breast (4.0%), hematological (3.1%), and others (3.6%)
Atterman et al. (24)	Observational cohort	Swedish Patient register	8228	75.1/both	NA	Warfarin	-	Major or NMCR bleeding, intracranial bleeding, and gastrointestinal bleeding	1.0	Prostate (27.2%), gastrointestinal (19.1%), pancreatic (1.0%), lung (6.8%), breast (9.1%), gynecological (4.9%), urological (35.6%), intracranial (1.3%), hematological (10.7%), metastasized (9.2%), and others (14.4%)
Chan et al. (21)	Observational cohort	Taiwan National Health Insurance Research Database	7955	77/both	dabigatran, rivaroxaban, apixaban, and edoxaban	Warfarin	SSE, VTE, and MI	Major bleeding, intracranial bleeding, and gastrointestinal bleeding	1.45	Not available

*AF, atrial fibrillation; DOACs, direct oral anticoagulants; VKAs, vitamin K antagonists; RCT, randomized controlled trial; SSE, stroke or systemic embolism; MI, myocardial infarction; VTE, venous thromboembolism; NMCR, non-major clinically relevant bleeding*.

### Crude Event Rates Between DOACs vs. VKAs

**Figure 1:**
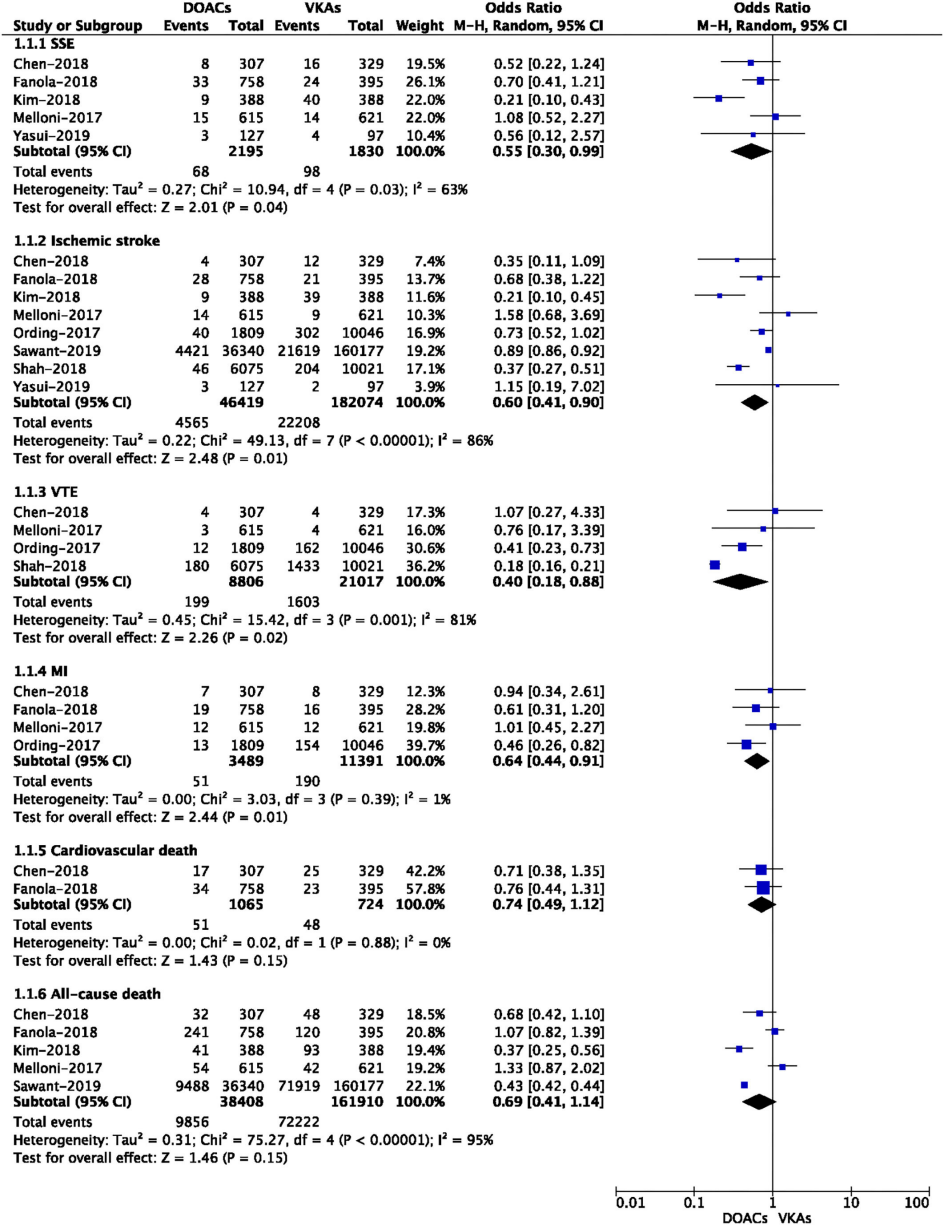
Crude effectiveness event rates of direct oral anticoagulants compared with vitamin K antagonists among atrial fibrillation patients with cancer.

A total of nine included studies reported the crude rates of effectiveness or safety outcomes between DOACs vs. VKAs (3, 4, 12–17, 19). For the effectiveness outcomes shown in Figure 1, compared with VKA-users, DOAC-users had lower event rates of SSE (3.10 vs. 5.36%, OR = 0.55, 95% CI: 0.30–0.99), ischemic stroke (9.83 vs. 12.2%, OR = 0.60, 95% CI: 0.41–0.90), VTE (2.26 vs. 7.63%, OR = 0.40, 95% CI: 0.18–0.88), and MI (1.46 vs. 1.67%, OR = 0.64, 95% CI: 0.44–0.91), but there were comparable rates of cardiovascular death (4.79 vs. 6.63%, OR = 0.74, 95% CI: 0.49–1.12) and all-cause death (25.7 vs. 44.6%, OR = 0.69, 95% CI: 0.41–1.14).

**Figure 2:**
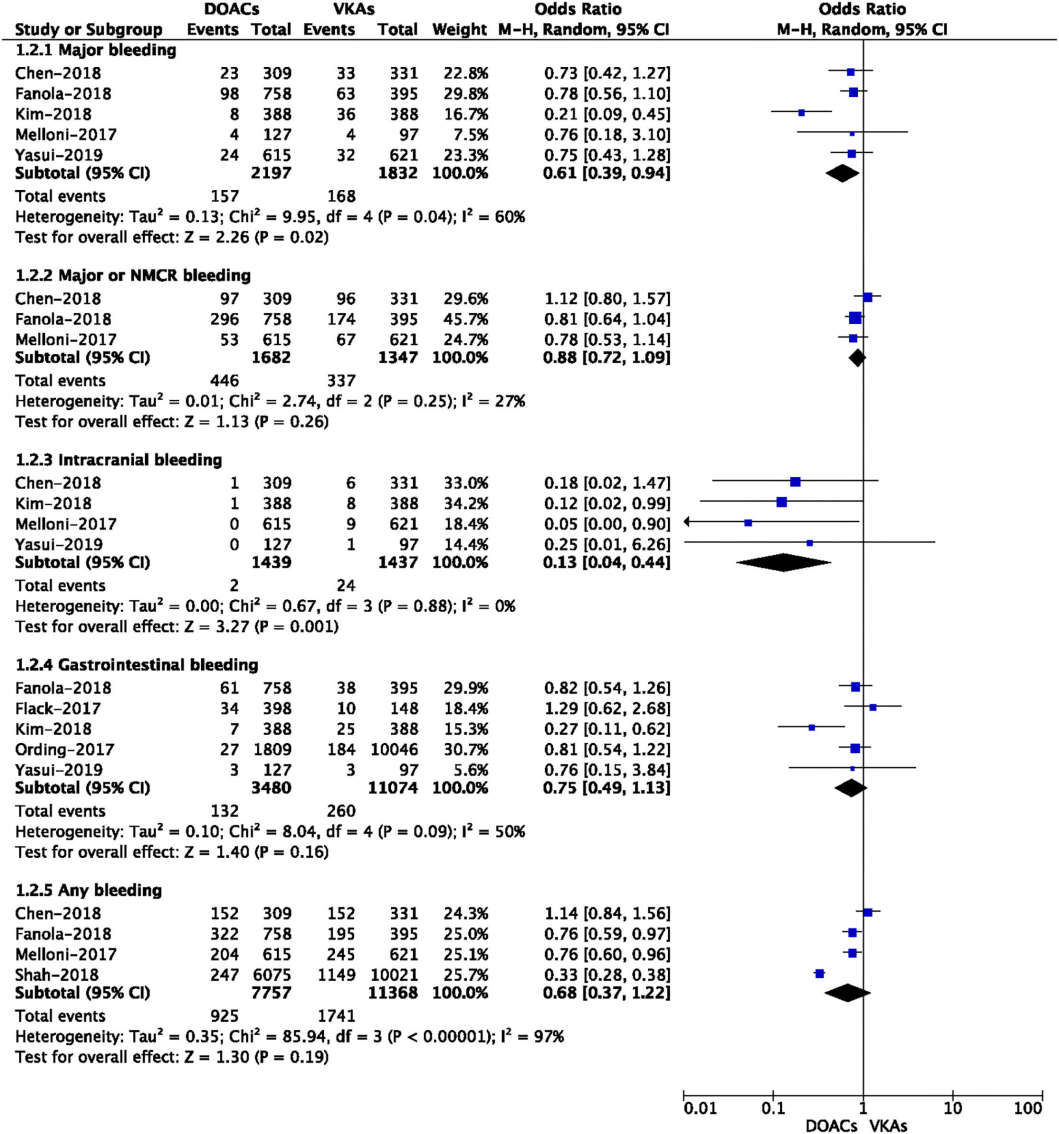
Crude safety event rates of direct oral anticoagulants compared with vitamin K antagonists among atrial fibrillation patients with cancer.

The safety outcomes of DOACs vs. VKA are presented in Figure 2. The pooled results showed that DOAC-users had lower event rates of major bleeding (7.15 vs. 9.17%, OR = 0.61, 95% CI: 0.39–0.94) and intracranial bleeding (0.14 vs. 1.67%, OR = 0.13, 95% CI: 0.04–0.44) than VKA-users. However, there were no significant differences in major or NMCR bleeding (26.5 vs. 25.0%, OR = 0.88, 95% CI: 0.72–1.09), gastrointestinal bleeding (3.79 vs. 2.34%, OR = 0.75, 95% CI: 0.49–1.13), and any bleeding (11.9 vs. 15.3%, OR = 0.68, 95% CI: 0.37–1.22) between the two studied groups.

### Adjusted Data of Outcomes Between DOACs vs. VKAs

**Figure 3:**
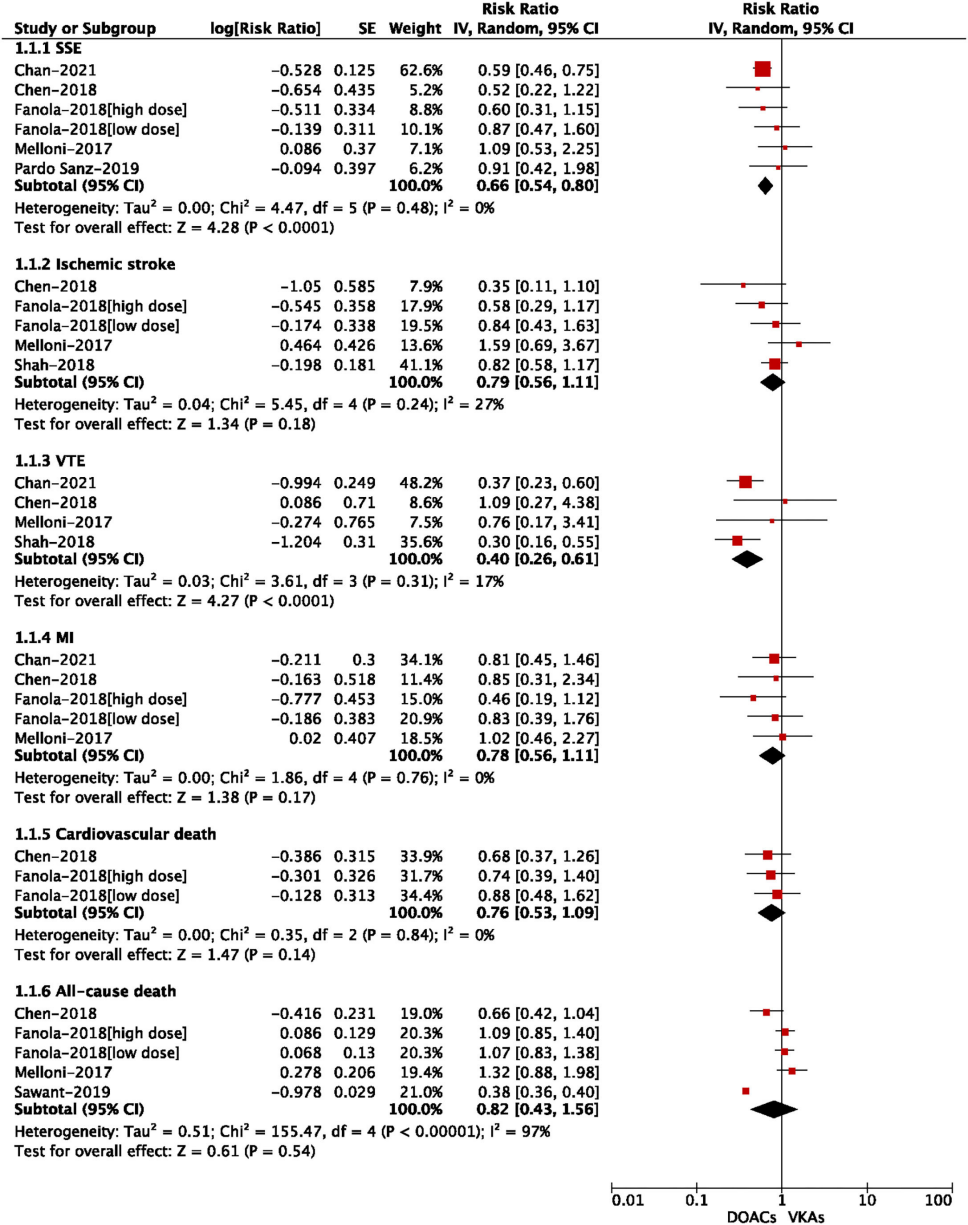
Adjusted effectiveness data of direct oral anticoagulants compared with vitamin K antagonists among atrial fibrillation patients with cancer.

A total of nine included studies reported the adjusted data of effectiveness or safety outcomes between DOACs vs. VKAs (3, 12–14, 17, 21–24). Adjusted confounders of the included studies are presented in Supplementary Table 2. As shown in Figure 3, for the effectiveness outcomes, the use of DOACs compared with VKA use was significantly associated with decreased risks of SSE (RR = 0.66, 95% CI: 0.54–0.80) and VTE (RR = 0.40, 95% CI: 0.26–0.61), but not ischemic stroke (RR = 0.79, 95% CI: 0.56–1.11), MI (RR = 0.78, 95% CI: 0.56–1.11), cardiovascular death (RR = 0.76, 95% CI: 0.53–1.09), and all-cause death (RR = 0.82, 95% CI: 0.43–1.56).

**Figure 4:**
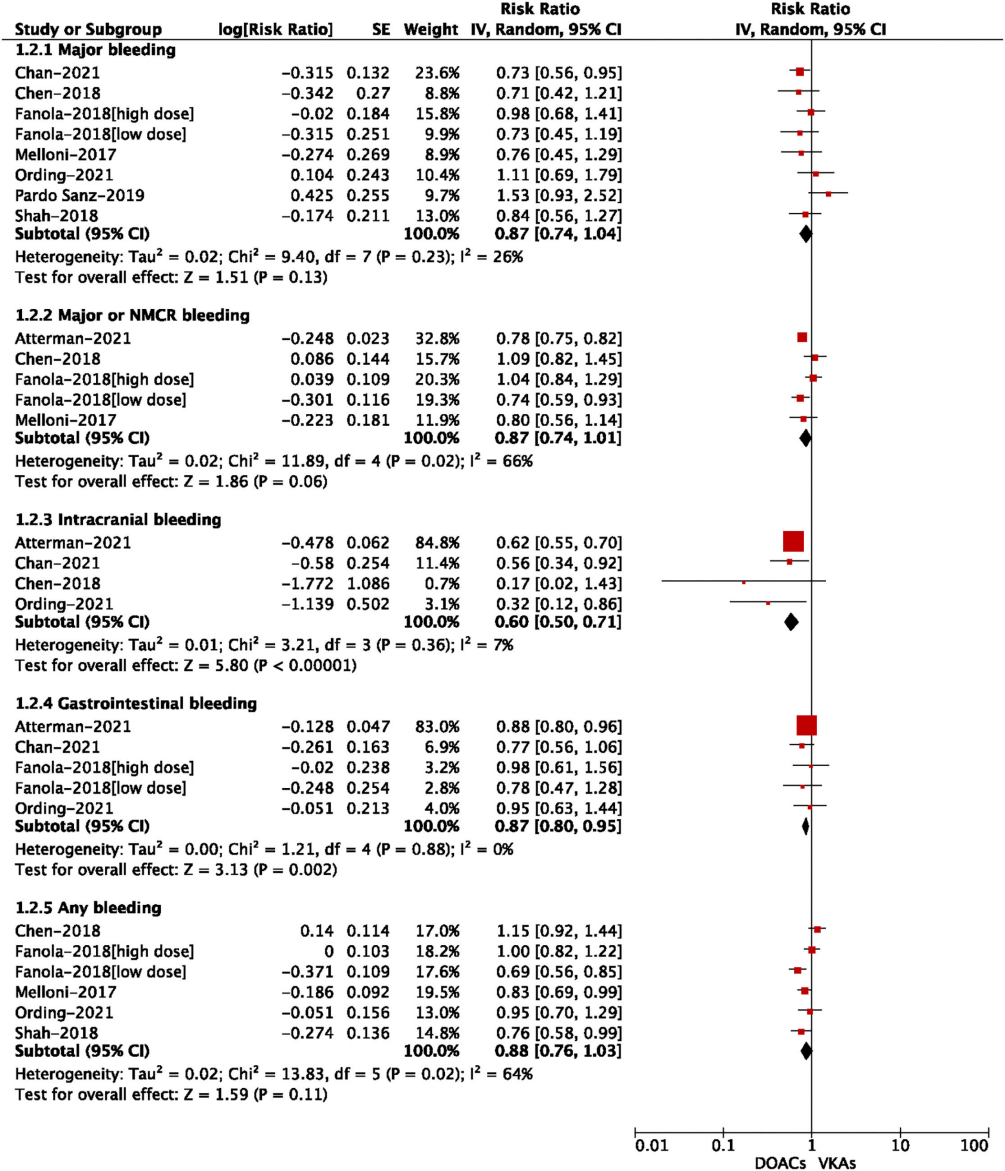
Adjusted safety data of direct oral anticoagulants compared with vitamin K antagonists among atrial fibrillation patients with cancer.

For the safety outcomes shown in Figure 4, compared with VKA use, the use of DOACs was significantly associated with reduced risks of intracranial bleeding (RR = 0.60, 95% CI: 0.50–0.71) and gastrointestinal bleeding (RR = 0.87, 95% CI: 0.80–0.95). There were no significant differences in major bleeding (RR = 0.87, 95% CI: 0.74–1.04), major or NMCR bleeding (RR = 0.87, 95% CI: 0.74–1.01), and any bleeding (RR = 0.88, 95% CI: 0.76–1.03).

### Publication Bias

As shown in Supplementary Figures 2, 3, no obvious publication biases were observed when assessed by using the funnel plots. Also, it was noted that the publication bias should not be evaluated for some reported outcomes when fewer than 10 included studies were included.

## Discussion

The main findings of our current study were as follows: (1) DOACs use resulted in lower rates of SSE and VTE as compared to VKAs use; (2) DOACs were associated with safer profiles (lower intracranial or gastrointestinal bleeding) than VKAs; (3) In comparison to VKAs, DOACs were non-inferior regarding the outcomes of ischemic stroke, MI, cardiovascular death, all-cause death, major bleeding, major or NMCR bleeding, and any bleeding.

Considering that malignant tumors have unique clinical risk characteristics, the optimal anticoagulant treatment for patients with AF and cancer is still controversial. On the one hand, cancer is a pro-thrombotic state, and further increases the risk of thromboembolism in patients with AF and cancer (25). On the other hand, cancer patients have a higher incidence of VTE and arterial thrombosis due to inflammatory cytokines, tumor vascular invasion, and vascular toxicity cancer treatments, while cancer-related thrombocytopenia and chemotherapy-related bone marrow suppression can increase bleeding complications (26–28). Not only that, some malignancies (e.g. primary or metastatic intracranial tumors and hematological malignancies) itself increase the risk of hemorrhage, potentially constituting contraindications to anticoagulation therapy or requiring thorough clinical surveillance even in patients at high thromboembolic risk. Therefore, concerns about bleeding complications and paucity of evidence-based data may result in the underuse of DOACs in cancer patients with AF.

Due to the extremely limited data, there are still no specific recommendations on the use of DOACs for cancer patients in the AF guidelines. Current RCTs involving antithrombotic therapy for cancer patients to prevent VTE have been published, the guidelines prefer LMWH over VKAs or DOACs in the prevention and treatment of VTE (5). Mounting evidence is demonstrating that DOACs could represent a valid choice in patients with cancer. Prior trials have shown that rivaroxaban and edoxaban are not inferior to LMWH in the treatment of cancer-related VTE (29, 30). Therefore, DOACs (rivaroxaban and edoxaban) are currently recommended for the treatment of VTE as an alternative treatment for LMWH in cancer patients (16, 31, 32). However, due to the different pathophysiology and risk characteristics between cancer and AF, these recommendations cannot be generalized to patients with cancer and AF.

Compared with DOACs, VKAs have several limitations, such as frequent international normalized ratio (INR) control, frequent dose adjustments, and diet or drug interactions. These deficiencies may be amplified in cancer and AF patients. In particular, chemotherapy drugs and warfarin have a strong pharmacological interaction, and cancer patients often have liver dysfunction, mucositis, or diarrhea, which lead to fluctuations in vitamin K absorption and increase the risk of anticoagulation therapy (33). Only about 12% of cancer patients receiving warfarin can obtain a stable INR therapeutic range (34). In addition, the anticoagulant activity of VKAs depends on TTR (time in therapeutic range). As such, it is difficult for cancer patients to receive cancer treatment to obtain the best INR range, and the prevalence of active cancer patients with TTR > 60% during the follow-up is only 10% (35). Moreover, DOACs are still more effective and safer than VKAs in AF patients with the best TTR (4).

The effectiveness and safety of DOACs compared with VKAs in AF and cancer patients have been explored in several recent studies. A prior systematic review by Russo et al. (36) supported that the effectiveness and safety profiles of NOACs in AF patients with malignancy appeared to be similar to those of VKA treatment. Unfortunately, they could not conduct a meta-analysis with the quantitative method to draw further conclusions due to the small number of included studies (36). Although the effectiveness and safety of DOACs and VKAs in AF patients with cancer are controversial, the conclusions seem to be more clear due to the emergence of several *post-hoc* analyses of RCTs and observational studies. Casula et al. (37) performed a meta-analysis by including three *post-hoc* analyses of RCTs (12–14), suggesting that direct oral Xa inhibitors (rivaroxaban, apixaban, edoxaban) had similar effects but were safer compared with warfarin in patients with cancer and AF. In addition to *post-hoc* analyses of RCTs, the meta-analyses by Chen et al. (38) and Mariani et al. (39) also included the different number of observational studies. By comparison, the largest number of studies (four *post-hoc* analyses of RCTs and nine observational cohorts) were included in our current meta-analysis. In addition, we assessed both crude event rates and adjusted data of outcomes between DOACs vs. VKAs in AF patients with cancer. Overall, in comparison to VKAs, DOACs appeared to have significant reductions in SSE, venous thromboembolism, intracranial bleeding, and gastrointestinal bleeding, but showed comparable rates of ischemic stroke, MI, cardiovascular death, all-cause death, major bleeding, major or NMCR bleeding, and any bleeding. Our meta-analysis was the largest and latest study comparing the effectiveness and safety outcomes of DOACs vs. VKAs in patients with non-valvular AF and cancer, potentially suggesting that DOACs might be considered suitable anticoagulant agents in this special population. Further prospective trials evaluating the effectiveness and safety of DOACs vs. VKAs in patients with AF combined with cancer could confirm our findings.

### Limitations

Our research still had some limitations. First, the clinical characteristics of patients in different included studies were heterogeneous, such as cancer type, cancer stage, cancer diagnosis time, anti-tumor drug use, or chemotherapy response. The incidence of thrombotic events varied with cancer types, stages, and patient-related or treatment-related factors. Second, all types of DOACs were analyzed together as one group despite their different pharmacological properties and differences in clinical effectiveness and safety in the different indications. Due to limited data, we did not conduct a subgroup analysis based on the specific types of DOACs. Third, we did not conduct a subgroup analysis of DOACs and VKAs between patients with active cancer and those with a history of cancer. Finally, data of RCTs and observational studies should be assessed separately in future studies.

## Conclusion

Current pooled data from the published studies suggested that in comparison to VKAs, DOACs appeared to have significant reductions in SSE, venous thromboembolism, intracranial bleeding, and gastrointestinal bleeding, but showed comparable rates of ischemic stroke, MI, cardiovascular death, all-cause death, major bleeding, major or NMCR bleeding, and any bleeding in patients with AF and cancer.

## Data Availability Statement

The original contributions presented in the study are included in the article/Supplementary Material, further inquiries can be directed to the corresponding author/s.

## Author Contributions

All authors listed have made a substantial, direct and intellectual contribution to the work, and approved it for publication.

## Funding

This study was funded by National Natural Science Foundation of China (82100273), China National Postdoctoral Program for Innovative Talents (BX20200400), and China Postdoctoral Science Foundation (2020M673016).

## Conflict of Interest

The authors declare that the research was conducted in the absence of any commercial or financial relationships that could be construed as a potential conflict of interest.

## Publisher's Note

All claims expressed in this article are solely those of the authors and do not necessarily represent those of their affiliated organizations, or those of the publisher, the editors and the reviewers. Any product that may be evaluated in this article, or claim that may be made by its manufacturer, is not guaranteed or endorsed by the publisher.

## References

[1] Hindricks G Potpara T Dagres N Arbelo E Bax JJ Blomstrom-Lundqvist C . 2020 ESC Guidelines for the diagnosis management of atrial fibrillation developed in collaboration with the European Association for Cardio-Thoracic Surgery (EACTS): the Task Force for the diagnosis management of atrial fibrillation of the European Society of Cardiology (ESC) Developed with the special contribution of the European Heart Rhythm Association (EHRA) of the ESC. Eur Heart J. (2021). 42:373–498. 10.1093/eurheartj/ehab648 32860505

[2] Chu G Versteeg HH Verschoor AJ Trines SA Hemels M Ay C . Atrial fibrillation and cancer - An unexplored field in cardiovascular oncology. Blood Rev. (2019) 35:59–67. 10.1016/j.blre.2019.03.005 30928168

[3] Shah S Norby FL Datta YH Lutsey PL MacLehose RF Chen LY . Comparative effectiveness of direct oral anticoagulants and warfarin in patients with cancer and atrial fibrillation. Blood Adv. (2018) 2:200–9. 10.1182/bloodadvances.2017010694 29378726 PMC5812321

[4] Kim K Lee Y Kim T Uhm J Pak H Lee M . Effect of non-vitamin K antagonist oral anticoagulants in atrial fibrillation patients with newly diagnosed cancer. Korean Circ J. (2018) 48:406. 10.4070/kcj.2017.0328 29671285 PMC5940645

[5] Hu YF Liu CJ Chang PM Tsao HM Lin YJ Chang SL . Incident thromboembolism and heart failure associated with new-onset atrial fibrillation in cancer patients. Int J Cardiol. (2013) 165:355–7. 10.1016/j.ijcard.2012.08.036 22989607

[6] Barrios V Escobar C Calderon A Rodriguez RG Llisterri JL Polo GJ. Use of antithrombotic therapy according to CHA2DS2-VASc score in patients with atrial fibrillation in primary care. Rev Esp Cardiol (Engl Ed). (2014) 67:150–1. 10.1016/j.rec.2013.07.009 24795129

[7] Kearon C Akl EA Ornelas J Blaivas A Jimenez D Bounameaux H . Antithrombotic therapy for VTE disease: CHEST Guideline and Expert Panel Report. CHEST. (2016) 149:315–52. 10.1016/j.chest.2015.11.026 26867832

[8] Giugliano RP Ruff CT Braunwald E Murphy SA Wiviott SD Halperin JL . Edoxaban versus warfarin in patients with atrial fibrillation. N Engl J Med. (2013) 369:2093–104. 10.1056/NEJMoa1310907 24251359

[9] Granger CB Alexander JH McMurray JJ Lopes RD Hylek EM Hanna M . Apixaban versus warfarin in patients with atrial fibrillation. N Engl J Med. (2011) 365:981–92. 10.1056/NEJMoa1107039 21870978

[10] Patel MR Mahaffey KW Garg J Pan G Singer DE Hacke W . Rivaroxaban versus warfarin in nonvalvular atrial fibrillation. N Engl J Med. (2011) 365:883–91. 10.1056/NEJMoa1009638 21830957

[11] Connolly SJ Ezekowitz MD Yusuf S Eikelboom J Oldgren J Parekh A . Dabigatran versus warfarin in patients with atrial fibrillation. N Engl J Med. (2009) 361:1139–51. 10.1056/NEJMoa0905561 19717844

[12] Chen ST Hellkamp AS Becker RC Berkowitz SD Breithardt G Fox K . Efficacy and safety of rivaroxaban vs. warfarin in patients with non-valvular atrial fibrillation and a history of cancer: observations from ROCKET AF. Eur Heart J Qual Care Clin Outcomes. (2019) 5:145–52. 10.1093/ehjqcco/qcy040 30219887

[13] Fanola CL Ruff CT Murphy SA Jin J Duggal A Babilonia NA . Efficacy and safety of edoxaban in patients with active malignancy and atrial fibrillation: analysis of the ENGAGE AF-TIMI 48 trial. J Am Heart Assoc. (2018) 7:e008987. 10.1161/JAHA.118.008987 30369307 PMC6201390

[14] Melloni C Dunning A Granger CB Thomas L Khouri MG Garcia DA . Efficacy and safety of apixaban versus warfarin in patients with atrial fibrillation and a history of cancer: insights from the ARISTOTLE trial. Am J Med. (2017) 130:1440–8. 10.1016/j.amjmed.2017.06.026 28739198

[15] Flack KF Desai J Kolb JM Chatterjee P Wallentin LC Ezekowitz M . Major gastrointestinal bleeding often is caused by occult malignancy in patients receiving warfarin or dabigatran to prevent stroke and systemic embolism from atrial fibrillation. Clin Gastroenterol Hepatol. (2017) 15:682–90. 10.1016/j.cgh.2016.10.011 27765728

[16] Ording AG Horváth Puhó E Adelborg K Pedersen L Prandoni P Sørensen HT. Thromboembolic and bleeding complications during oral anticoagulation therapy in cancer patients with atrial fibrillation: a Danish nationwide population-based cohort study. Cancer Med. (2017) 6:1165–72. 10.1002/cam4.1054 28544489 PMC5463075

[17] Sawant AC Kumar A Mccray W Tetewsky S Parone L Sridhara S . Superior safety of direct oral anticoagulants compared to Warfarin in patients with atrial fibrillation and underlying cancer: a national veterans affairs database study. J Geriatr Cardiol. (2019) 16:706–9. 10.11909/j.issn.1671-5411.2019.09.006 31645857 PMC6790962

[18] Wu VC Wang CL Huang YT Lan WC Wu M Kuo CF . Novel Oral Anticoagulant versus Warfarin in cancer patients with atrial fibrillation: an 8-year population-based cohort study. J Cancer. (2020) 11:92–9. 10.7150/jca.36468 31892976 PMC6930400

[19] Yasui T Shioyama W Oboshi M Oka T Fujita M. Oral anticoagulants in Japanese patients with atrial fibrillation and active cancer. Intern Med. (2019) 58:1845–9. 10.2169/internalmedicine.2415-18 30799369 PMC6663538

[20] Zhu W Ye Z Chen S Wu D He J Dong Y . Comparative effectiveness and safety of non-vitamin K antagonist oral anticoagulants in atrial fibrillation patients. Stroke. (2021) 52:1225–33. 10.1161/STROKEAHA.120.031007 33596677

[21] Chan Y Chao T Lee H Chen S Li P Liu J . Clinical outcomes in atrial fibrillation patients with a history of cancer treated with non-vitamin K antagonist oral anticoagulants: a Nationwide Cohort Study. Stroke. (2021) 52:3132–4. 10.1161/STROKEAHA.120.033470 34233467

[22] Ording AG Søgaard M Skjøth F Grove EL Lip GYH Larsen TB . Bleeding complications in patients with gastrointestinal cancer and atrial fibrillation treated with oral anticoagulants. Cancer Med US. (2021) 10:4405–14. 10.1002/cam4.4012 34114733 PMC8267127

[23] Pardo Sanz A Rincón LM Guedes Ramallo P Belarte Tornero LC de Lara Delgado G Tamayo Obregon A . Current status of anticoagulation in patients with breast cancer and atrial fibrillation. Breast. (2019) 46:163–9. 10.1016/j.breast.2019.05.017 31220790

[24] Atterman A Friberg L Asplund K Engdahl J. Atrial fibrillation, oral anticoagulants, and concomitant active cancer: benefits and risks. TH Open. (2021) 05:e176–e82. 10.1055/s-0041-1728670 34104856 PMC8169314

[25] Falanga A Marchetti M Russo L. The mechanisms of cancer-associated thrombosis. Thromb Res. (2015) 135(Suppl. 1):S8–S11. 10.1016/S0049-3848(15)50432-5 25903541

[26] Piazza G. Venous thromboembolism and cancer. Circulation. (2013) 128:2614–8. 10.1161/CIRCULATIONAHA.113.002702 24344060

[27] Aronson D Brenner B. Arterial thrombosis and cancer. Thromb Res. (2018) 164(Suppl. 1):S23–S8. 10.1016/j.thromres.2018.01.003 29703480

[28] Kamphuisen PW Beyer-Westendorf J. Bleeding complications during anticoagulant treatment in patients with cancer. Thromb Res. (2014) 133(Suppl. 2):S49–S55. 10.1016/S0049-3848(14)50009-6 24862146

[29] Raskob GE van Es N Verhamme P Carrier M Di Nisio M Garcia D . Edoxaban for the treatment of cancer-associated venous thromboembolism. N Engl J Med. (2018) 378:615–24. 10.1056/NEJMoa1711948 29231094

[30] Young AM Marshall A Thirlwall J Chapman O Lokare A Hill C . Comparison of an oral factor Xa inhibitor with low molecular weight heparin in patients with cancer with venous thromboembolism: results of a randomized trial (SELECT-D). J Clin Oncol. (2018) 36:2017–23. 10.1200/JCO.2018.78.8034 29746227

[31] Kraaijpoel N Carrier M. How I treat cancer-associated venous thromboembolism. Blood. (2019) 133:291–8. 10.1182/blood-2018-08-835595 30478093

[32] Agnelli G Becattini C Meyer G Munoz A Huisman MV Connors JM . Apixaban for the treatment of venous thromboembolism associated with cancer. N Engl J Med. (2020) 382:1599–607. 10.1056/NEJMoa1915103 32223112

[33] Rose AJ Sharman JP Ozonoff A Henault LE Hylek EM. Effectiveness of warfarin among patients with cancer. J Gen Intern Med. (2007) 22:997–1002. 10.1007/s11606-007-0228-y 17476542 PMC2219727

[34] Vranckx P Valgimigli M Heidbuchel H. The significance of drug-drug and drug-food interactions of oral anticoagulation. Arrhythm Electrophysiol Rev. (2018) 7:55–61. 10.15420/aer.2017.50.1 29636974 PMC5889806

[35] Lee YJ Park JK Uhm JS Kim JY Pak HN Lee MH . Bleeding risk and major adverse events in patients with cancer on oral anticoagulation therapy. Int J Cardiol. (2016) 203:372–8. 10.1016/j.ijcard.2015.10.166 26539960

[36] Russo V Bottino R Rago A Micco PD D' OA Liccardo B . Atrial fibrillation and malignancy: the clinical performance of non-vitamin K oral anticoagulants-a systematic review. Semin Thromb Hemost. (2019) 45:205–14. 10.1055/s-0038-1661386 30119139

[37] Casula M Fortuni F Fabris F Leonardi S Gnecchi M Sanzo A . Direct oral Xa inhibitors versus warfarin in patients with cancer and atrial fibrillation: a meta-analysis. J Cardiovasc Med (Hagerstown). (2020) 21:570–6. 10.2459/JCM.0000000000001041 32628422

[38] Chen Y Mao M Chang J Yan J Yang T Liu Y . Safety and efficacy of new oral anticoagulants compared to those of warfarin in AF patients with cancer: a meta-analysis of randomized clinical trials and observational studies. Eur J Clin Pharmacol. (2021) 77:849–57. 10.1007/s00228-021-03132-x 33791828

[39] Mariani MV Magnocavallo M Straito M Piro A Severino P Iannucci G . Direct oral anticoagulants versus vitamin K antagonists in patients with atrial fibrillation and cancer a meta-analysis. J Thromb Thrombolysis. (2021) 51:419–29. 10.1007/s11239-020-02304-3 33044735 PMC7886836

