# Supplementary material for: Effectiveness and Safety of DOACs vs. VKAs in AF Patients With Cancer: Evidence From Randomized Clinical Trials and Observational Studies
Source: Front Cardiovasc Med. 2021 Nov 5;8:766377. doi: 10.3389/fcvm.2021.766377 (PMC8602680; doi:10.3389/fcvm.2021.766377)
Supplement: Supplementary file 1 [file Data_Sheet_1.PDF]

Supplementary Table 1. The search strategies of this meta-analysis

	Search terms	No.
**PubMed**		
#1	atrial fibrillation	92116
#2	cancer OR tumor OR malignancy	2021342
#3	non-vitamin K antagonist oral anticoagulants OR direct oral anticoagulants OR dabigatran OR rivaroxaban OR apixaban OR edoxaban	16047
#4	vitamin K antagonists OR warfarin	38425
#5	#1 and #2 and #3 and #4	172
**Embase**		
#1	atrial fibrillation	189562
#2	cancer	4588663
#3	tumor	3247476
#4	malignancy	278446
#5	#2 OR #3 OR #4	5975476
#6	non-vitamin K antagonist oral anticoagulants	1397
#7	direct oral anticoagulants	6104
#8	dabigatran	18172
#9	rivaroxaban	21502
#10	apixaban	15321
#11	edoxaban	5982
#12	#6 OR #7 OR #8 OR #9 OR #10 OR #11	35873
#13	vitamin K antagonists	7703
#14	warfarin	101846

#15	#13 OR #14	106755
#16	#1 and #5 and #12 and #15	944

Supplementary Table 2. Adjusted confounders of the included studies

**Included studies** / **Confounders**	**Chen-2018**	**Fanola-2018**	**Melloni-2017**	**Ording-2021**	**Shah-2018**	**Pardo Sanz-2019**	**Sawant-2019**	**Atterman-2021**	**Chan-2021**
Age	√	√	√	√	√		√	√	√
Gender	√	√		√	√		√	√	
Geographic region	√	√	√						
Race		√							
**Clinical assessment**									
CHA2DS2-VASc					√	√			
HAS-BLED						√			
Body mass index	√								
Weight			√						
Smoking		√							
Alcohol	√							√	
Fall								√	
Cancer type				√		√	√		
AF type	√		√						
High-dimensional					√				

propensity scores									
Albumin	√								
Platelets	√								
Kidney function	√	√						√	
**Comorbid conditions/past medical history**									
Ischaemic heart disease		√							√
Heart failure	√	√		√				√	√
Diabetes mellitus	√	√	√	√				√	√
Hypertension	√	√	√	√				√	√
Anemia	√							√	
Vascular diseases	.		√					√	
TIA/stroke/systemic embolism	√	√	√	√	√				√
History of bleeding	√			√	√			√	√
Hypothyroidism		√							√
Renal disease									√
Liver disease								√	√
Chronic lung disease	√								√
Peripheral artery disease		√							

**Medications**									
Anticoagulant s(VKAs, etc,)	√	√	√			√		√	
ARB/ACE-I									√
Calcium channel blocker									√
Beta-blockers									√
Platelet inhibitors	√								√
Lipid lowering drugs				√					√
H2 blockers									√
NSAIDS									√

Note: The included studies of Ording-2017,Flack-2017,Kim-2018, and Yasui-2019 were used for analysis regarding the crude rates of effectiveness or safety outcomes between DOACs versus VKAs.

ARB/ACE-I=angiotensin receptor blocker/angiotensin converting enzyme inhibitor; NSAIDs=non-steroid anti-inflammatory drugs.

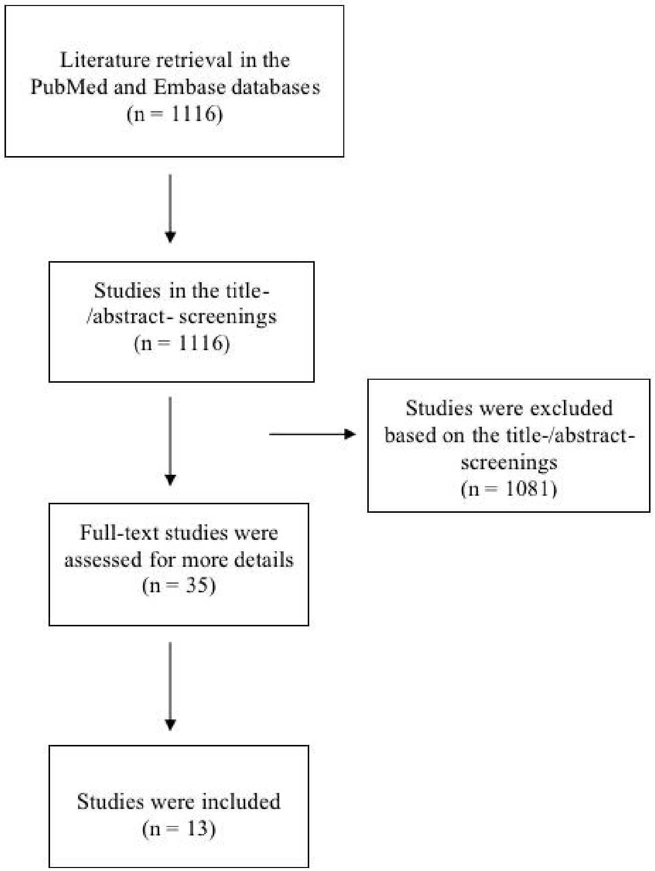

Supplementary Figure 1. The process of the literature retrieval of this meta-analysis

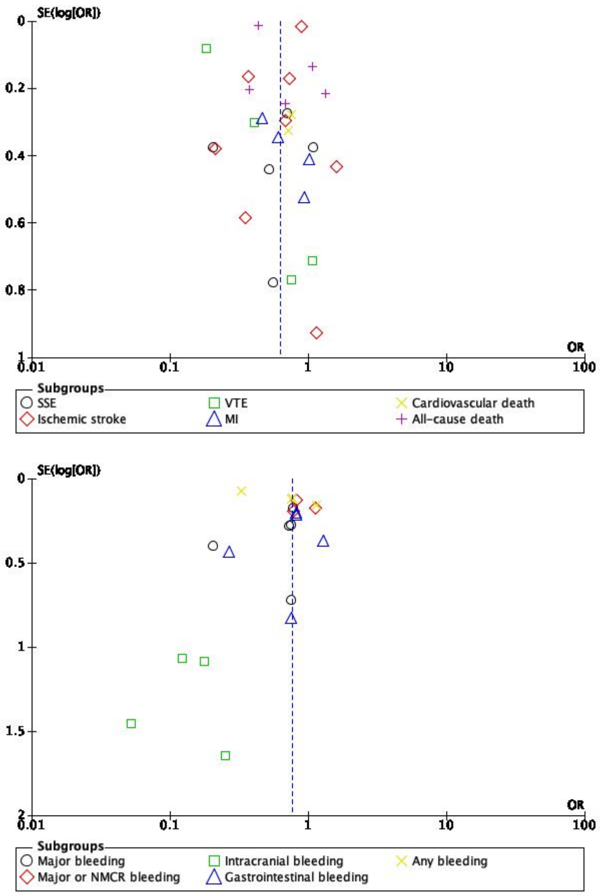

Supplementary Figure 2. The funnel plots for effectiveness and safety outcomes of DOACs versus warfarin (unadjusted data)

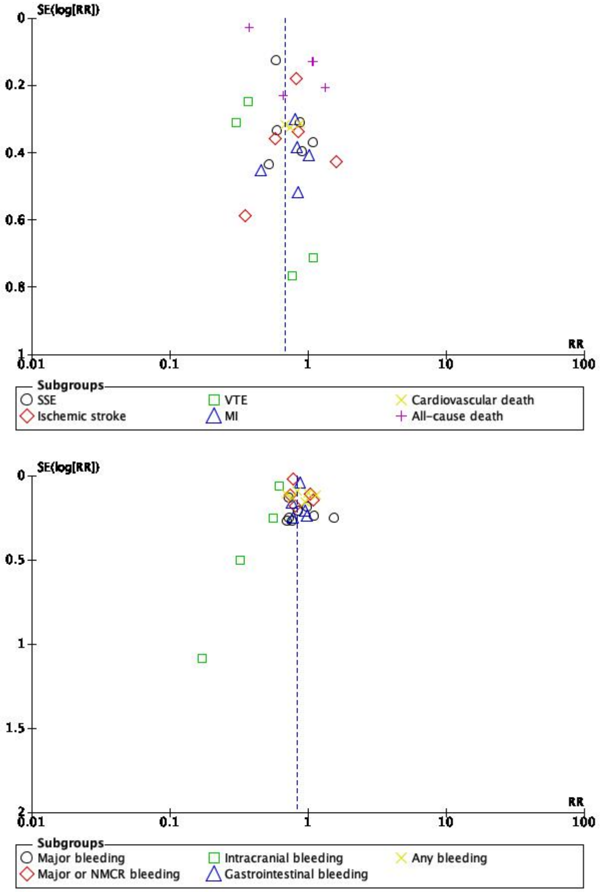

Supplementary Figure 3. The funnel plots for effectiveness and safety outcomes of DOACs versus warfarin (adjusted data)
